# Beyond Simpson's Rule: Accounting for Orientation and Ellipticity Assumptions

**DOI:** 10.1016/j.ultrasmedbio.2022.07.013

**Published:** 2022-12

**Authors:** Woo-Jin Cho Kim, Arian Beqiri, Adam J. Lewandowski, Esther Puyol-Antón, Deborah C. Markham, Andrew P. King, Paul Leeson, Pablo Lamata

**Affiliations:** ⁎ School of Biomedical Engineering and Imaging Sciences, King's College London, London, UK; † Ultromics Ltd, Oxford, UK; ‡ Cardiovascular Clinical Research Facility, Division of Cardiovascular Medicine, University of Oxford, Oxford, UK

**Keywords:** Two-dimensional echocardiography, Left ventricle volumes, Modified Simpson's biplane rule, Apical chamber views

## Abstract

Simpson's biplane rule (SBR) is considered the gold standard method for left ventricle (LV) volume quantification from echocardiography but relies on a summation-of-disks approach that makes assumptions about LV orientation and cross-sectional shape. We aim to identify key limiting factors in SBR and to develop a new robust standard for volume quantification. Three methods for computing LV volume were studied: (i) SBR, (ii) addition of a truncated basal cone (TBC) to SBR and (iii) a novel method of basal-oriented disks (BODs). Three retrospective cohorts representative of the young, adult healthy and heart failure populations were used to study the impact of anatomical variations in volume computations. Results reveal how basal slanting can cause over- and underestimation of volume, with errors by SBR and TBC >10 mL for slanting angles >6°. Only the BOD method correctly accounted for basal slanting, reducing relative volume errors by SBR from –2.23 ± 2.21% to –0.70 ± 1.91% in the adult population and similar qualitative performance in the other two cohorts. In conclusion, the summation of basal oriented disks, a novel interpretation of SBR, is a more accurate and precise method for estimating LV volume.

## Introduction

Information on ventricular morphology and function is of utmost importance for the diagnosis of numerous heart diseases (Baicu et al. 2005). Left ventricle (LV) volumes and ejection fraction (LVEF) have a major prognostic value in predicting adverse outcomes for patients with heart failure (White et al. 1987; Hwang et al. 1989; Wong et al. 1993; Bonhorst et al. 2019). Although cardiac magnetic resonance (CMR) imaging is considered the gold standard for LV quantification, 2-D echocardiography (2DE) remains the first-line choice because of its cost-efficiency and widespread availability (Bellenger 2000; Malm et al. 2004; Ciampi and Villari 2007).

Left ventricle volumes are commonly calculated using the modified Simpson's biplane rule (SBR), which relies on endocardial border delineations of two longitudinal acquisition planes (Grossgasteiger et al. 2014). SBR, a summation-of-disks method, is however limited by geometric assumptions regarding LV shape. First, the longitudinal planes are assumed to be perpendicular and aligned to the axes of the elliptical cross-section of the LV. And second, the basal plane is considered perpendicular to these longitudinal planes. Commercial solutions thereby alter the SBR formulation to alleviate the impact of these assumptions. These modifications are neither detailed in the literature nor accounted for in cross-center studies and, thus, are a source of uncontrolled variability in clinical practice.

The recommended longitudinal planes for LV tracings are the apical four-chamber (A4C) and two-chamber (A2C) views (Lang et al. 2015). The A4C is standard in most echocardiographic protocols as it is the easiest and most reproducible to perform because the aortic and mitral valves serve as anatomical landmarks (Romanovsky et al. 1991; Feigenbaum 1996). There is, however, a debate over which orthogonal view conforms best with the biplane rule. Although the American Society of Echocardiography (ASE) recommends the A2C for this matter (Lang et al. 2015), there is evidence to suggest improved accuracy of biplane measurements with the apical three-chamber long-axis view (A3C or APLAX) (Nosir et al. 1997; Malm et al. 2005).

In this work, we present a study of the impact of the LV geometrical assumptions on the accuracy and robustness of SBR with the aim of defining the methodological refinements that best address these assumptions. The study of which orthogonal view is better, A2C or A3C, is a second objective.

## Methods

### Simpson's bi-plane rule

**Fig. 1:**
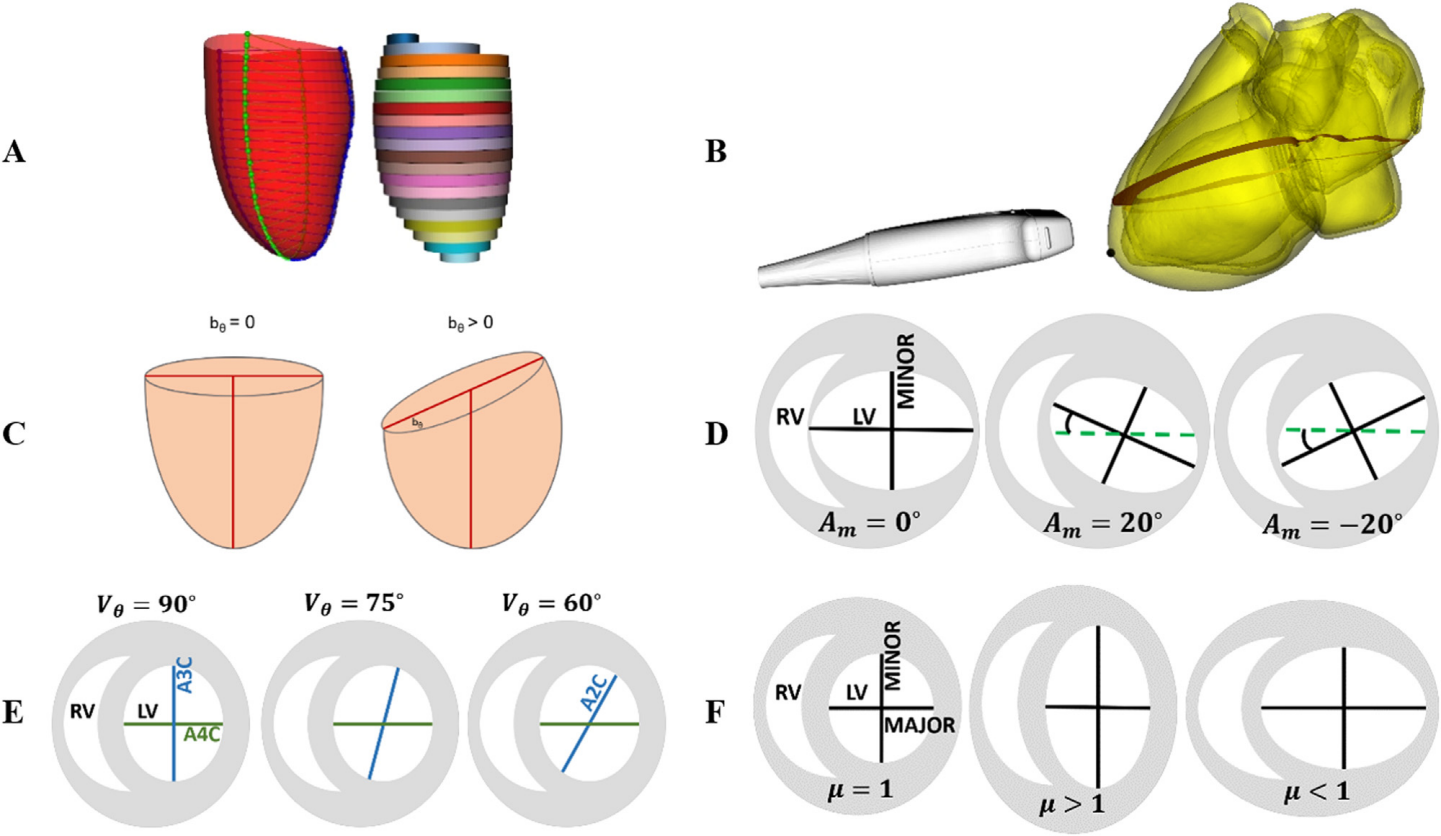
Definition of Simpson's biplane rule (SBR) and parametrization of the acquisition and anatomical assumptions that lead to errors in volume computations. (a) Schematic of SBR, the summation-of-disks method from two longitudinal planes. (b) Foreshortening that leads to volume underestimation (*red plane* = foreshortened view, *black dot* = apex). (c) Basal slanting that is parametrized with angle $b_{\theta }$ and leads to volume underestimation. (d) Planes not capturing the axes of the elliptical cross-sections, parametrized with the orientation angle *A_m_*, leading to a varying range of volume over- and underestimations (*green line* = A4C, *blue line* = A2C, *pink line* = direction of major axis). (e) Varying viewing angles $v_{\theta }$ gradually from A3D ($90^{\circ }$) to A2C ($60^{\circ }$). (f) Varying eccentricities μ where a value of 1 corresponds to a perfect circle. LV = left ventricle; RV = right ventricle.

The definition of the summation-of-disks method (Lang et al. 2015) is described as the process of dividing the LV cavity into multiple cylinders of equal height (Fig. 1a) where the volume of each individual disk V is estimated as

(1) $$V=\;\frac{\pi \left(a\times b\right)L}{4n}$$

where a and b are the major and minor axis diameters, L is the length of the LV cavity and n is the total number of disks. Once the volumes for each individual disk are obtained, the total LV volume $V_{T}$ is computed using

(2) $$V_{T}=\;\frac{\pi L}{4n}\sum_{i=1}^{n}a_{i}\times b_{i}$$

In clinical practice informed by the ASE guidelines (Schiller et al. 1989), $a_{i}$ and $b_{i}$ represent the A4C and A2C contour diameters of the *i*th disk and n=20 in SBR.

### Limitations in SBR caused by acquisition difficulties and anatomical assumptions

The most severe limitation during acquisition is the presence of foreshortening (Jenkins et al. 2008), a specific case of probe misalignment where the ultrasound plane fails to cut through the apex (Fig. 1b). The LV cavity in these cases appears shorter and may cause an underestimation in volume.

Anatomical assumptions are another source of errors. Although SBR works sufficiently well for simple LV geometries with little to no heterogeneity, it does not perform robustly under severe anatomical variations (Myhr et al. 2018). First, SBR assumes that the basal plane is perfectly perpendicular to the apicobasal direction that defines the disks, but LV anatomies have different degrees of basal slanting, which occurs when the basal plane is no longer perpendicular to the long axis in one or both views simultaneously. This will be a source of underestimation with SBR in the slanted basal region dependent on the angle of slanting $b_{\theta },$ as illustrated in Figure 1c.

The second anatomical assumption of SBR is that all cross-sectional slices of the LV cavity are perfect ellipses whose main axes are contained in the longitudinal planes of image acquisition (*e.g.,* A4C and A2C). This assumption can be mathematically formulated by three parameters (Fig. 1d–f): the orientation angle $A_{m}$ of the first longitudinal plane (*i.e.,* the A4C) with respect to the true major axis of the ellipse of each cross section, the viewing angle $v_{\theta }\;$between the two longitudinal planes (*e.g.,* A4C and A2C) and the eccentricity μ defined as the ratio between the major and minor axes of each cross section. For the SBR formulation to work correctly, $A_{m}$ should be 0° or 90° and $v_{\theta }$ should be 90° so that the views are capturing the major and minor axes of the ellipse. Any deviation will cause errors whose magnitude depends on the eccentricity μ (the more eccentric, the larger the error).

### Strategies to cope with acquisition and anatomical limitations

#### TBC and BOD methods

The elliptic assumption on the LV cross-section is a bold yet necessary requirement for a bi-plane volume estimation task. We have only two vertical longitudinal contours with which to estimate LV volume. The inherent inaccuracies from such assumptions are inevitable owing to the limited dimensionality of the data (Myhr et al. 2018).

The impact of foreshortening is reduced by choosing the long-axis dimension L in eqn (2) as the largest between the two views (Mercier et al. 1982). This solution mitigates the underestimation when only one of the views is foreshortened, but it will not correct if both views suffer from this acquisition limitation.

**Fig. 2:**
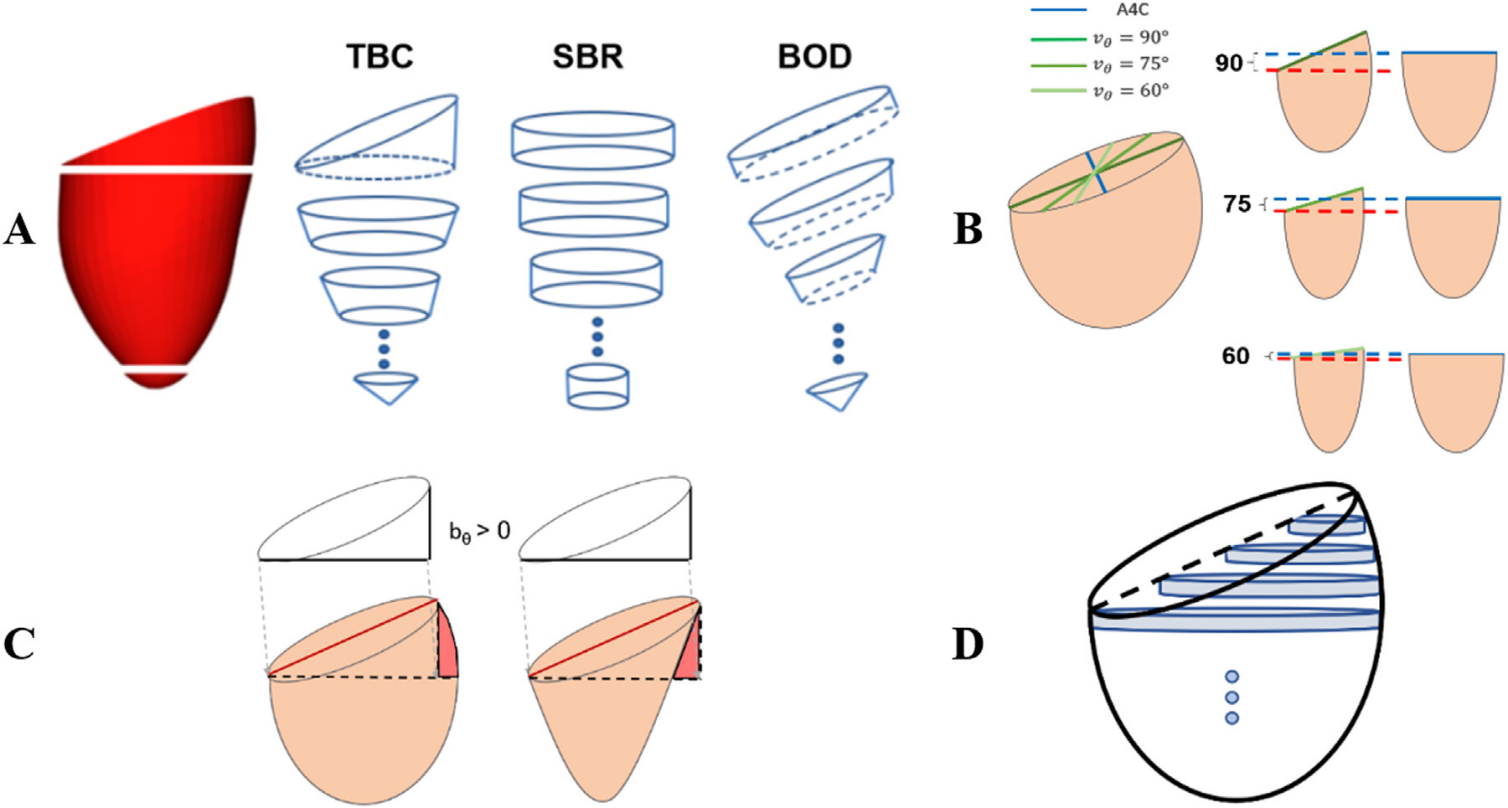
(a) Schematic of the Simpson disk stacking for truncated basal cylinder (TBC), Simpson's bare rule (SBR) and basal-oriented-disks (BOD) methods. (b) Overestimation with TBC caused by choosing the largest L (*blue dotted line*) rather than the correct length L is represented by the *red dotted line*. (c) Variable errors with TBC depending on the curvature of the LV wall, where the truncated basal cylinder to approximate the basal region leads to either an overestimation (left) or underestimation (right). (d) SBR underestimation caused by poor fitting of cylindrical disks around the slanted base. The *dotted lines* represent the top of the basal plane of each view—see Figure S3B (online only) for an illustration in a real left ventricle anatomy.

On the other hand, basal slanting has been addressed in the literature by adding one extra half-cut cylinder in the basal region (Santamore et al. 1973), a strategy that constitutes the core characteristic of the truncated basal cylinder (TBC) method illustrated in Figure 2a. This truncated cylinder causes overestimation of volume if the two vertical views do not have the same angle of slanting $b_{\theta }$ because the long-axis dimension L in the less slanted view will be an overestimation (Fig. 2b). TBC also suffers from a variable under- or over-estimation of volume if lateral walls are not straight, as illustrated in Figure 2c. As a more robust alternative to account for basal slanting, in this work we propose a novel approach, the orientation of the disks according to the basal plane, which defines the basal oriented disk (BOD) method (Fig. 2a).

There are two other strategies that aim to improve the accuracy of volumes, which in this work are included in both TBC and BOD methods. First each disk is tailored with two (top and bottom) estimated cross-sections, instead of a single mid-cross-section; in other words, each disk is approximated by using an elliptical frustrum rather than a cylinder (Santamore et al. 1973). And second, the last disk in the apex is replaced by an elliptical cone. Elliptical frustrums and the apical cone better represent the anatomy and the changes along the LV walls’ curvature.

### Evaluation workbenches

First, synthetic LV paraboloids, with idealized elliptical cross sections as assumed by all methods, are used to study the correction of the anatomical assumptions. Paraboloids were generated with varying $A_{m}$ and μ but with no slice-to-slice variability in these two cross-sectional characteristics. Basal slanting was generated by cropping the paraboloids with a cutting plane at angles $b_{\theta }$ ranging from 0° to 20°. Two different configurations of the synthetic paraboloids were generated, where the A4C and basal slanting planes were aligned with either the true major or minor axes ($A_{m}=0^{\circ }\;\text{and}\;90^{\circ }$ respectively).

The second evaluation workbench is a set of three cohorts of 3-D LV anatomies reconstructed from clinical MRI data sets. The main reference is a healthy subset of the UKBiobank (Ruijsink et al. 2020) (UKBB) database (exclusion criteria: participants with self-reported cardiovascular condition), with 4113 participants (2062 males, 61.4 ± 7.5 y). To evaluate generality of findings and the impact of differences in anatomical characteristics across cohorts, two more cohorts are included: a young healthy cohort (YHC), with 225 participants (108 males, 26.4 ± 2.1 y) who were scanned to study the impact of premature birth (Lewandowski et al. 2013); and a heart failure cohort (HFC), with 50 participants (39 males, 69 ± 11.2 y) with HF who were selected for cardiac resynchronization therapy (Warriner et al. 2018). Population demographics are found in Table S1 (online only).

The YHC and UKBB 3-D LV anatomies are built from semi-automatic segmentations (one observer for YHC and fully automatic for UKBB [Lewandowski et al. 2013]) of steady-state free precession (SSFP) short-axis stack (SAX) acquisitions, and the HFC anatomies, from manual segmentations (consensus between two observers) of 3-D SSFP acquisitions (Warriner et al. 2018). Accordingly, YHC and UKBB anatomies are truncated below the valve plane as present in SAX slices.

The 3-D anatomies are built using methods that are robust to acquisition and segmentation errors by using computational meshes with smooth basis functions (Lamata et al. 2011, 2014). The normalized vector direction from the LV center to the RV center ($RV_{\text{dir}})$ is accessible for all populations by segmentation of the RV blood pool.

The volume of the blood pool of the 3-D generated meshes, from both the idealized and the patient-specific cases, becomes the ground truth for all computations. To generate apical views for volume estimation, 2-D slices were sampled from the anatomical meshes to mimic echocardiography images. The long axis is defined as the line connecting the apex and the mitral valve center. The A4C slices were extracted by performing a 18° counterclockwise rotation around the long axis from the $RV_{\text{dir}}$ vector (additional details are given in the Supplementary Data, online only). Subsequently, the A2C and A3C slices were extracted by further 60° and 90° counterclockwise rotations, respectively, around the long axis from the A4C plane (Schiller et al. 1989). All slices were generated through the Visualization Tool Kit (VTK) Python library.

## Results

### Study on synthetic idealized anatomies

**Fig. 3:**
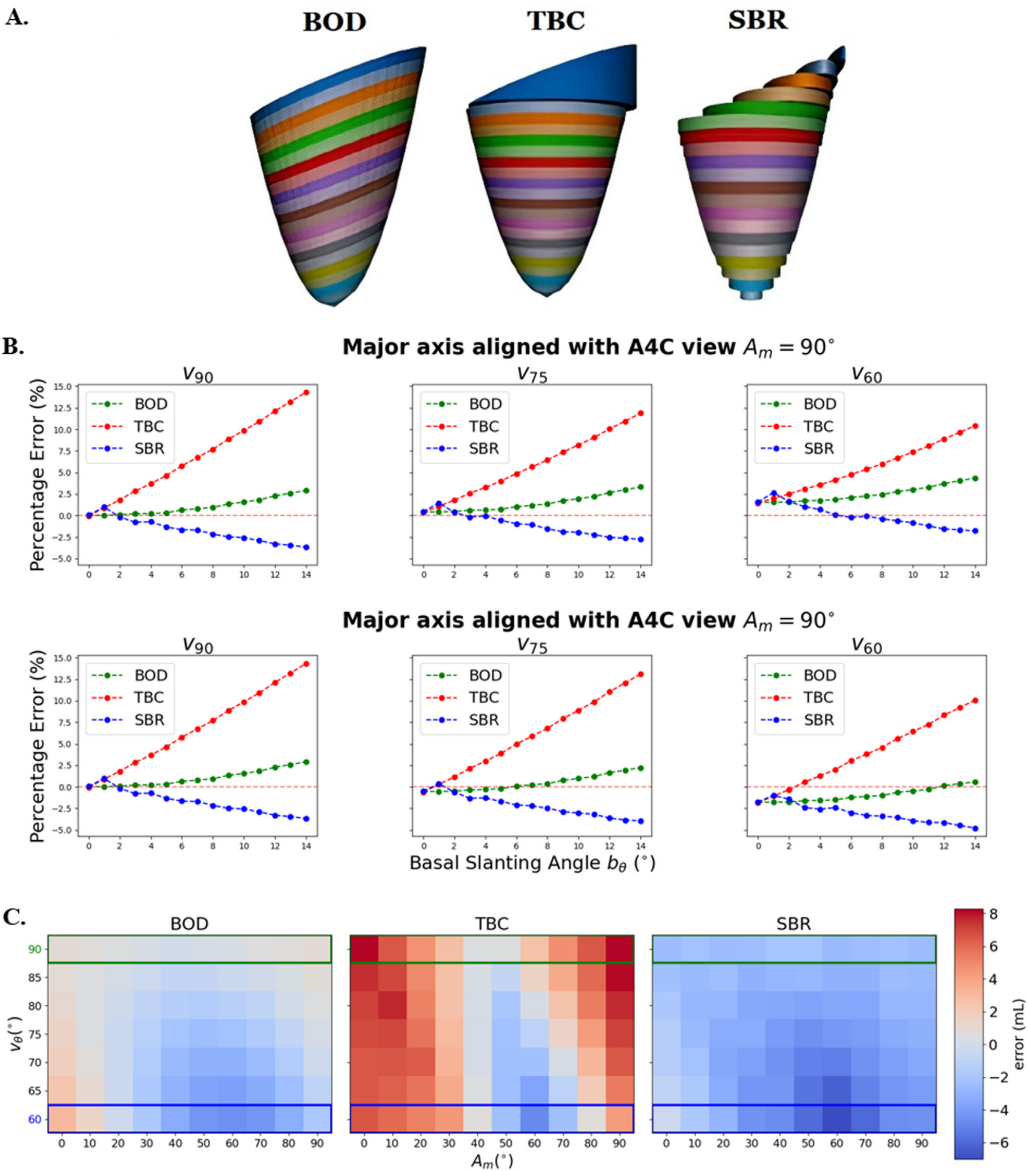
Performance of the three formulations (simple bare rule [SBR], truncated basal cylinder [TBC] and BOD: basal oriented disks [BODs]) on the synthetic data set. (a) Illustrative example of the disks formed by each formulation on a left ventricle represented by an ellipsoid with a large basal slanting angle $\left(b_{\theta }=20^{\circ }\right)$. (b) Error in volume estimation by the three formulations with varying basal slanting angles $\left(b_{\theta }=\left\{0^{\circ },\;20^{\circ }\right\}\right)$ in two orientation angles ($A_{m}=90^{\circ }\;\text{and}\;0^{\circ }$, top and bottom rows) and in three viewing angles ($v_{\theta }=90^{\circ },\;75^{\circ },60^{\circ }$, in columns) and eccentricity μ=1.1. (c) Relative error in volume computation with varying viewing ($v_{\theta })$ and orientation ($A_{m})$ angles, with fixed eccentricity µ = 1.1 and basal slanting $b_{\theta }=5^{\circ }$ for both views. Viewing angle of $v_{\theta }=60^{\circ }$ is representative of the angle between 4ch and 2ch views (highlighted by *blue rectangles* in panel C), and $v_{\theta }=90^{\circ }$ is representative of the angle between 4ch and A3C views (highlighted by *green rectangles* in panel C).

The BOD method outperforms TBC and SBR in the study of varying basal slanting angles with idealized viewing and orientation angles ($v_{\theta }=90^{\circ },\;A_{m}=0^{\circ }$) (Fig. 3b). For small slanting angles of $b_{\theta }<4^{\circ }$, all methods had percentage volume errors no greater than 5%. Yet, with increasing $b_{\theta }$, the BOD method yielded lower errors compared with the notable over- and under-estimation by TBC and SBR, respectively. For example, at $b_{\theta }=7^{\circ }$, the percentage errors for each method were 0.25% (BOD), 11.04% (TBC) and 4.28% (SBR).

The underestimation in SBR volumes corresponded to the elongated SBR disks formed by shorter diameters around the basal region, as illustrated in Figure 2a. The overestimation by TBC is explained by the factors illustrated in Figure 2b and 2c, the overestimation of long-axis length (L value, see Fig. 2b) being the most relevant. These errors are irrespective of eccentricity.

The BOD method is the most robust method to basal slanting across different viewing angles $v_{\theta }$ (Fig. 3), and all three methods suffer from the same systematic bias with changing $v_{\theta }$: volumes are underestimated when the major axis is missed (*e.g.,* $v_{\theta }<90^{\circ }$ and minor axis aligned with 4ch or $A_{m}=0^{\circ }$, top panel in Fig. 3b) and volumes are overestimated when the minor axis is missed (*i.e.,* $v_{\theta }<90^{\circ }$ and major axis aligned with 4ch or $A_{m}=90^{\circ }$, bottom panel in Fig. 3b). Accordingly, these biases with changing viewing angles $v_{\theta }$ are proportional to the degree of eccentricity (*i.e.,* no errors in circular cross-sections or μ=1).

Focusing on $v_{\theta }=60^{\circ }$, a reasonable estimate of the viewing angle between conventional 4ch and 2ch views, the TBC method will lead to the smallest bias with $A_{m}=90^{\circ }$ because of its compensation of two sources of error (overestimation caused by basal slanting and underestimation caused by missing the major axis) and to the largest bias with $A_{m}=0^{\circ }$ because of the addition of the same two sources of error.

The variable error in volume estimation depending on viewing angles $v_{\theta }$ and orientation angles $A_{m}$ is illustrated in Figure 3c, illustrating the more robust behavior of the BOD method. SBR volumes were mostly underestimated whereas TBC was overestimated with a maximum error of 10.8 mL. The TBC method had the highest variation in volume errors, peaking at $A_{m}=40^{\circ },\;50^{\circ }$ and $60^{\circ }$.

### Study on 3-D reconstructed anatomies from MRI

Among the UKBB volunteers, three participants were excluded from the analysis because of poor SAX alignment forming the 3-D reconstructed mesh. No more cases were excluded from any of the cohorts.

**Fig. 4:**
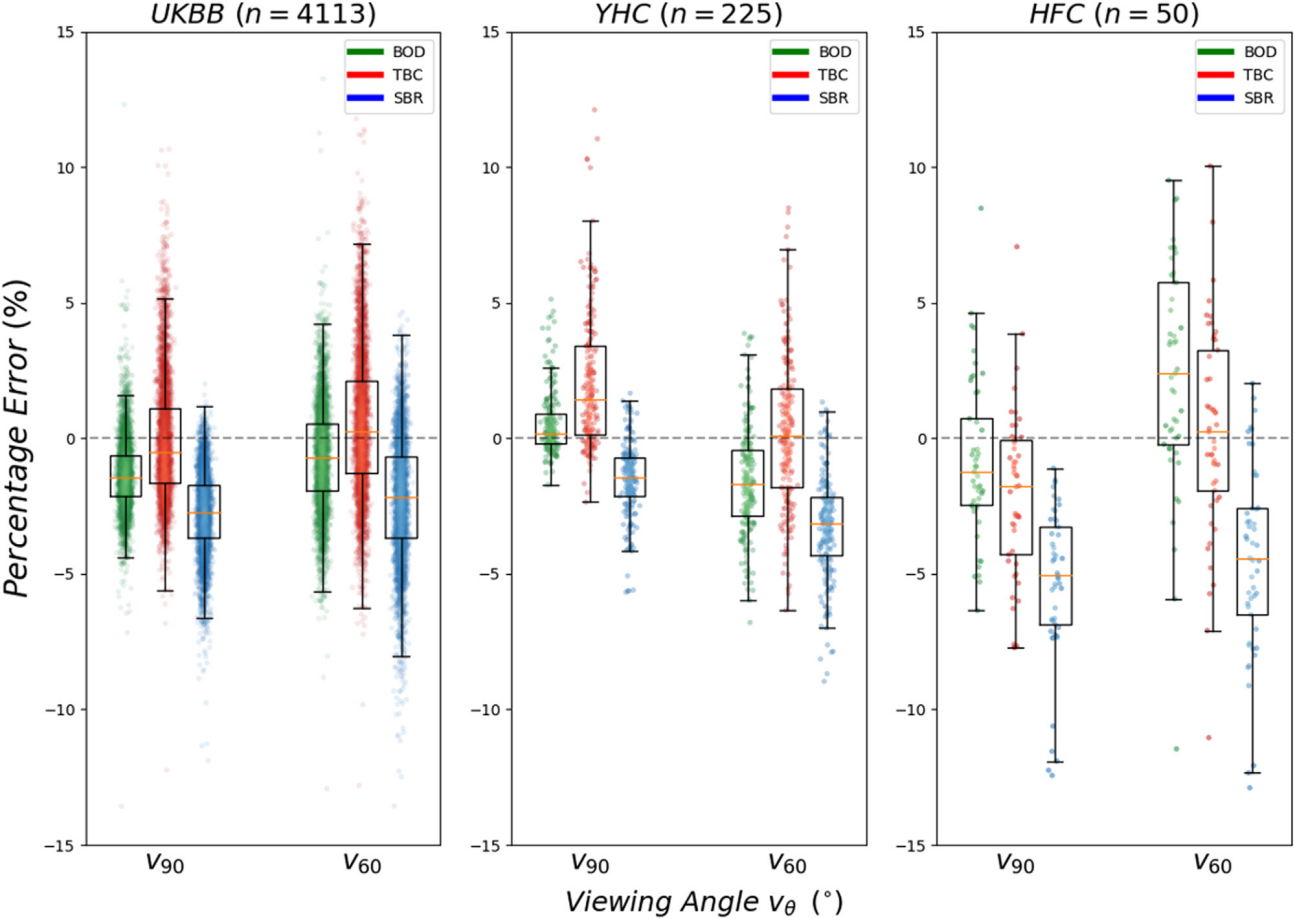
Percentage error in the estimation of left ventricle volume by the three methods (BOD, TBC, SBR) and in the viewing angles corresponding to the secondary view chosen as the A3C (V90) and A2c (V60), across the three cohorts (UKBB, YHC and HFC). BOD = basal oriented disk; HFC = heart failure cohort; SBR = Simpson's biplane rule; TBC = truncated basal cone; UKBB = UKBiobank (Ruijsink et al. 2020) (UKBB) database; YHC = young healthy cohort.

The SBR method was the most inaccurate method (*i.e.,* it had the largest bias), systematically underestimating LV volumes across the three cohorts and two viewing angles (average of –3.27%, peak at –5.35%). BOD and TBC methods were both more accurate, with a reduction in bias to averages of –0.26% and 0.14%, respectively. TBC was the most imprecise method with the largest standard deviation of errors and an average of 2.83% as compared with 2.20% and 2.22% for SBR and BOD, respectively (Fig. 4).

The lowest performance for all methods occurred at the viewing angle $v_{\theta }=60$. This corresponds to the use of the A2C view as the secondary view, where the BOD reduced relative volume errors by SBR from –2.23 ± 2.21% to –0.70 ± 1.91% in the UKBB cohort (see Table S2 [online only] for exact volume estimates and statistical analysis). Basal slanting had a different prevalence across the three cohorts (Fig. S7, online only): the percentages of cases in which $b_{\theta }>12^{\circ }$ in the A4C view were 10.4%, 21.1% and 58.0% in the UKBB, YHC and HFC populations, respectively. Furthermore, the degree of cross-sectional eccentricity was considerably higher in healthy participants than in patients with heart failure (Fig. S4, online only). Performance in volume computations was the worst for the HFC, matching this larger prevalence of basal slanting but being the cohort with smallest cross-sectional eccentricity.

The reconstructed LV anatomy exhibited high variability in ellipticity parameters $A_{m}$ and μ, not only across cases but also within each case over its cross-sectional slices (see the eccentricity profiles in Fig. S7).

## Discussion

To the best of our knowledge, this is the first study to evaluate the impact of anatomical variations in the computation of volumes by the summation-of-disks method. The conventional SBR is unable to accurately capture basal slanting, an issue that is not corrected with a TBC but with BODs. Any biplane formulation suffers from the variations in the orientation ($A_{m}$) and eccentricity (μ) of the cross sections in the human left ventricle that cannot be corrected for from two vertical planes. Finally, A3C is a more precise second view for volume computations than A2C because disk volumes are best estimated with perpendicular vertical planes.

The most relevant source of error in biplane volume estimation is foreshortening, which causes an underestimation of the long-axis length and, thus, of ventricular volume. Length must therefore be estimated from the longest of the two vertical planes to minimize the impact of this limitation. Basal slanting, causing an underestimation by SBR, is the second most relevant factor, the one that has been addressed in this work. The interplay between these two sources of error explains why existing TBC correction of SBR can cause a large volume overestimation: the basal slanted view requires a cut through part of the long-axis length to fit the truncated cylinder in the base, a length cut that will still be corrected as if it was caused by foreshortening (see an illustration of this effect in Fig. 2b). This is the main reason why the BOD formulation outperforms TBC both in synthetic and real anatomies.

The amount of basal slanting has been characterized in our 3 cohorts, revealing that it is a quite prevalent and a relatively homogeneous characteristic across them (Fig. S7). Basal slanting is a characteristic that manifests as an apicobasal tilting in 3-D statistical shape models of the LV shape, and has been reported to discriminate those born prematurely (Lewandowski et al. 2013) or to be a unique signature of aortic stenosis (Chan et al. 2019). The potential impact of using the adequate formulation can thus depend on the condition of the patient, and the benefit of BOD over TBC in selected diseased groups might be greater than that reported in this work.

The impact of TBC's overestimation caused by foreshortening correction depends mainly on the difference in basal slanting between the two vertical views. In the worst-case scenario, with only one view having basal slanting, it may lead to a larger overestimation than the underestimation of the bare SBR, with errors scaling up to 10% in presence of basal slanting angles of 7.5° that we have reported to be relatively common across our three populations (Fig. S7). The impact in real anatomies depends on the eccentricity and orientation of the cross-sections, where certain conformations may even lead TBC to cancel error factors and provide accuracy superior to that of BOD. Our study in three different cohorts found that TBC's average overestimation compared with BOD is small.

Current echocardiography guidelines recommend the use of the A2C as the orthogonal view for biplane measurements. However, previous studies have reported closer limits of agreement with 3DE volumes using A3C rather than A2C (Nosir et al. 1997; Malm et al. 2005). Our results, based on idealized angles of 60° and 90°, further support the use of A3C because it is a more perpendicular view with respect to A4C, and non-orthogonal viewing planes introduce a variable bias on the computed volumes. It is worth remarking that neither of these two views is exactly orthogonal to A4C. A study on 27 patients found that spatial angles for A4C–A2C and A4C–A3C are 63.3 ± 19.7° and 99.1 ± 25.6° respectively at end-diastole (Nosir et al. 1997).

Potential errors in volume computation depending on the viewing and orientation anglesare directly proportional to the eccentricity: the more eccentric (*i.e.,* μ less close to 1), the larger is the potential error (Teichholz et al. 1976; Kronik et al. 1979; Grothues et al. 2002). All three methods have the worst performance in the HFC, a cohort that displays the most circular cross-sections (*i.e.,* should have smaller errors) and the largest variability in major axis alignment (*i.e.,* should have larger error variability) compared with the other two cohorts (Figs. S5 and S6, online only). Accordingly, the variability in major axis alignment (*i.e.,* parameters $v_{\theta }$ and $A_{m}$) seems to be playing the biggest role in the large errors of this cohort.

Error in volume estimation could be reduced by using the patient's specific orientation angle and eccentricity, and one could hypothesize that an extra short-axis slice could provide these two parameters and lead to a patient-specific correction factor. This is an approach that was attempted and led to some improvement in accuracy at the cost of precision (results not reported here). One of the causes of this finding is that the human left ventricle exhibits huge variability in cross-sectional orientation angle and eccentricity, not only between persons but also within cross-sectional slices of each LV (Supplementary Data Sect. A4, online only). This result suggests that because of the large variation in cross-sectional morphology within each LV, an extra short-axis slice will still not be representative enough to account for the patient's specific orientation angle and eccentricity.

The relevance of the BOD method is underscored by the fact that slanted bases are prevalent even in healthy volunteers. In cases with large slanting angles (>15°), the use of BODs can lead to a correction in volume errors >15 mL compared with the biplane measurements provided by existing solutions. The BOD method improved accuracy and precision of SBR, reducing relative volume errors from –2.23 ± 2.21% to –0.70 ± 1.91% across the reference population of UKBB healthy participants and displaying a similar qualitative performance in the other two cohorts.

### Limitations

This work is subject to two main sources of limitations. First, we have not been able to report results with real echocardiographic views because precise and accurate ground truth volumes are challenging. Instead, we have defined and made available a large workbench of 3-D anatomies with ideal matching 2-D contours that remove any segmentation and cross-modality confounding factor in the study of the formulations that compute the volume of the left ventricle. And second, the 3-D geometries used in YHC and UKBB cohorts are truncated ellipsoids, as reconstructed from SAX MRI data, that miss a bit of the basal LV anatomy. This truncation should not affect the findings on cross-section orientations but could potentially affect the quantification of basal slanting: SAX MRI acquisitions are planned by the orientation of the valve planes, and we can only speculate that truncation should follow a consistent and congruent criterion that will lead to similar characteristics of basal slanting as compared with direct measurements in echocardiographic images.

### Clinical implications

Left ventricular volumes, and associated volumetric measures such as ejection fraction, are used to diagnose, monitor and manage most cardiac conditions. Small errors in left ventricular volumetric measures, particularly in conditions such as heart failure and valvular disease, where specific dimensions are used as thresholds for clinical decisions, can have significant implications for patients. For instance, if measurements indicate a patient has moved from moderate to severe left ventricular dysfunction, this may trigger consideration for interventions such as cardiac resynchronization (McDonagh et al. 2021). Similarly, evidence of left ventricular dilatation beyond a certain threshold is an indication for valve surgery in some conditions (Vahanian et al. 2022). As a result, additional imaging tests such as CMR may be ordered to increase precision of volumetric measures. By ensuring accurate measures with echocardiography these more expensive and additional techniques might be avoided.

## Conclusions

The BOD formulation with an A3C second view is the choice that leads to the most precise and accurate LV volume estimations.

## Appendix Supplementary materials

Image, application 1
