# Supplementary material for: Beyond Simpson's Rule: Accounting for Orientation and Ellipticity Assumptions
Source: Ultrasound Med Biol. 2022 Dec;48(12):2476–85. doi: 10.1016/j.ultrasmedbio.2022.07.013 (PMC9810537; doi:10.1016/j.ultrasmedbio.2022.07.013)
Supplement: Supplementary file 1 [file mmc1.docx]

# **Supplementary Material**

# **Beyond Simpson’s rule: accounting for orientation and ellipticity assumptions, by**

Woo-Jin Cho Kim_1_, Arian Beqiri_2_, Adam J. Lewandowski_3_, Esther Puyol_1_, Deborah Markham_2_, Andrew King_1_, Paul Leeson_3_, and Pablo Lamata_1_

## A1 Definition of the A4C orientation.

The A4C view is defined by the centre of valve planes, but this information was not available in our data. Instead, the centre of mass of the left and right ventricular cavities was available. The centre of mitral valve can be assumed to be aligned in vertical with the centre of mass of the left ventricle. Nevertheless, the centre of the tricuspid valve is not aligned with the centre of mass of the right ventricle, and the angular difference between these two references is estimated here.

The 3D cardiac anatomy of 20 control subjects (i.e. admitted to hospital with a false heart attack) was segmented and reconstructed from 3D computer tomography scans. We define the *RV_dir_* plane as the plane intersecting the apex, LV center and RV center, and the A4C plane as the one intersecting apex, LV center and tricuspid valve centre. The average angle between these two planes across the 20 subjects is found to be 17.9°±5.1.

| 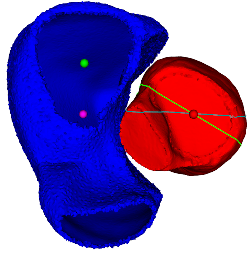 | 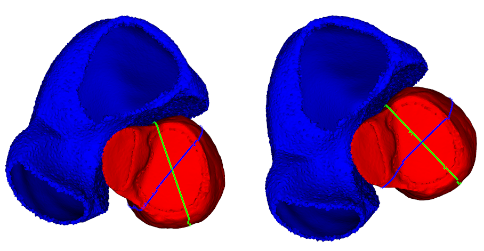 |
| --- | --- |
| (a) | (b) |

Fig. S1: Definition of the orientation of the A4C views based on the center of mass of the ventricles. (a) Top view of the ventricles, with the center of mass of the LV (red sphere), RV (pink sphere) and tricuspid valve (green sphere), and with the two vertical planes that set the counter-clockwise angle between the two planes averaged over the 20 CT SAX meshes is. (b) Illustration of the viewing angles $v_{60}$(left) and $v_{90}\left( right \right).$

## A2 Synthetic study: Results on varying *v_θ_* with no eccentricity.


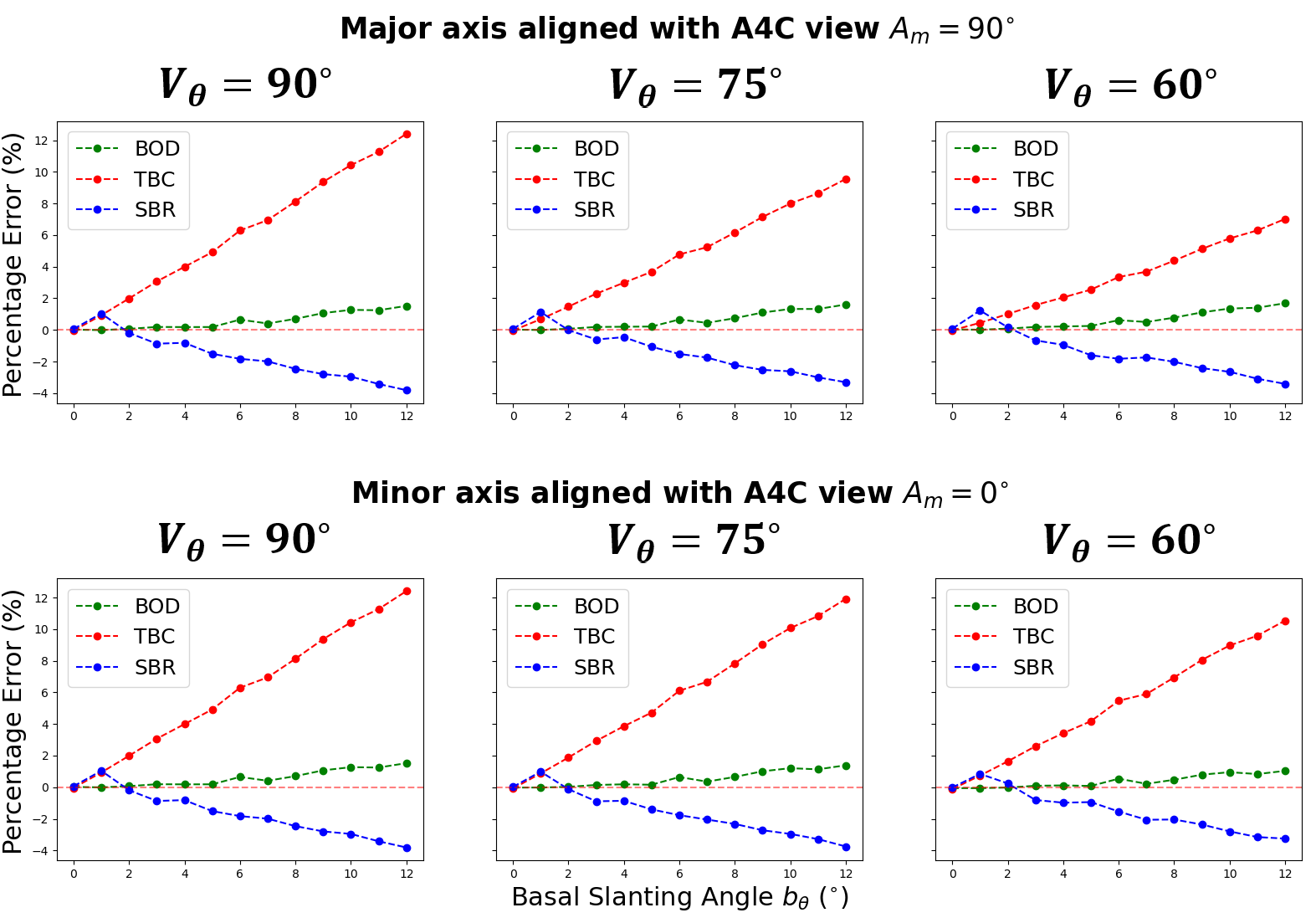


Fig. S2: Performance of SBR (magenta), TBC (blue), BOD (green) methods across different viewing angles $v_{\theta}=\left[ 90^{\circ}, 75^{\circ}, 60^{\circ} \right]$with eccentricity $\mu=1$ and with the two idealised orientation angles ($Am=0^{\circ}, 90$).

## A3 Illustration of SBR in one real anatomy

| 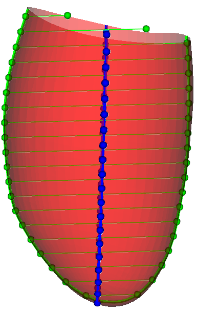 | 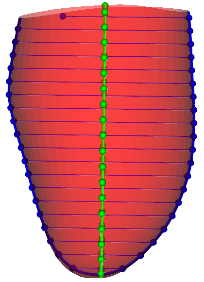 | 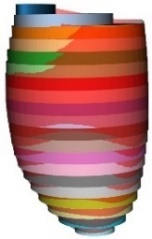 | 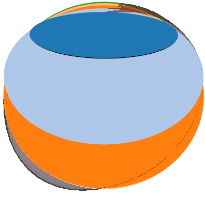 |
| --- | --- | --- | --- |
| (a) | (b) | (c) | (d) |

Fig. S3: SBR rule reveals that disks around the basal region do not encompass the true underlying anatomy. (a) A4C landmarks, (b) orthogonal view landmarks, (c) rendered SB disks, and (d) rendered SB disks from top-view (base to apex direction)

## A4 Eccentricity and slanting characteristics of clinical MRI meshes.

| 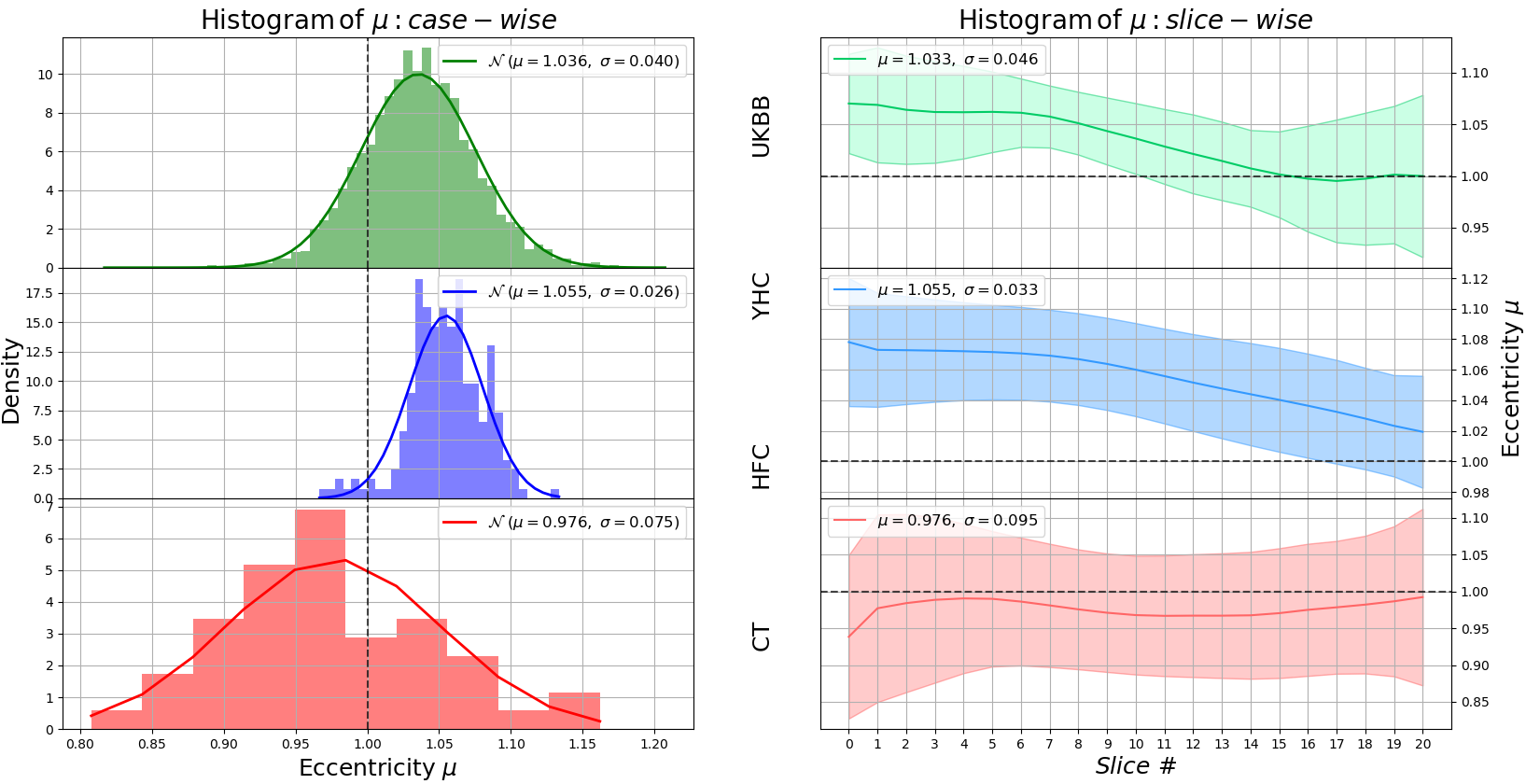 | |
| --- | --- |
| (a) | (b) |

Fig. S4: (a) Histogram of the case-wise eccentricity (μ) averaged across all slices, and (b) distribution (central line as mean, gray area as range covering +/- 1std) of the slice-wise eccentricity across all cases in each of the three populations. Slice number 0 corresponds to the basal slice.


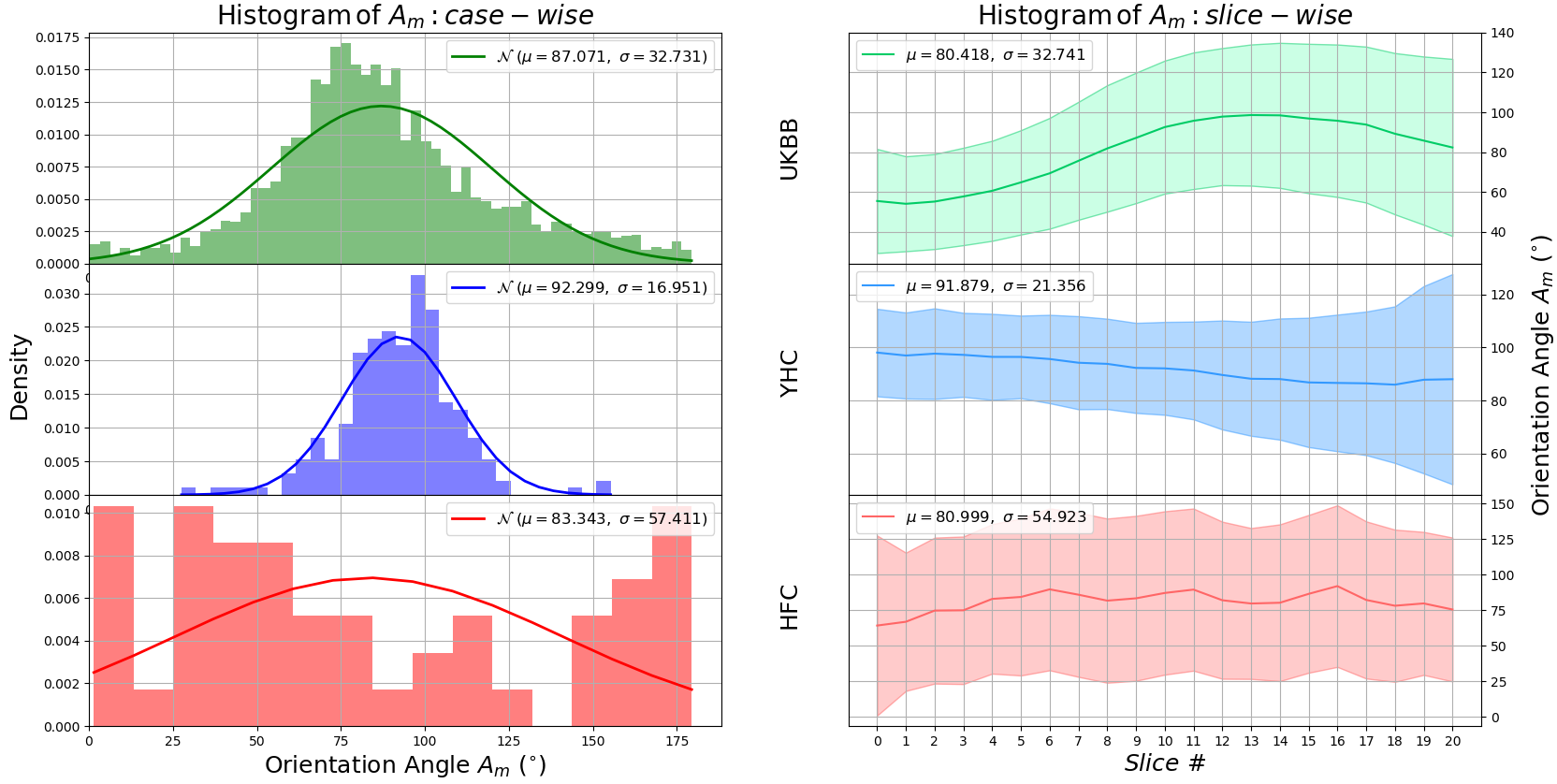


| (a) | (b) |
| --- | --- |

Fig. S5: (a) Histogram of the case-wise orientation angle (A_m_) averaged across all slices, and (b) distribution (central line as mean, gray area as range covering +/- 1std) of the slice-wise orientation angle across all cases in each of the three populations. Slice number 0 corresponds to the basal slice.


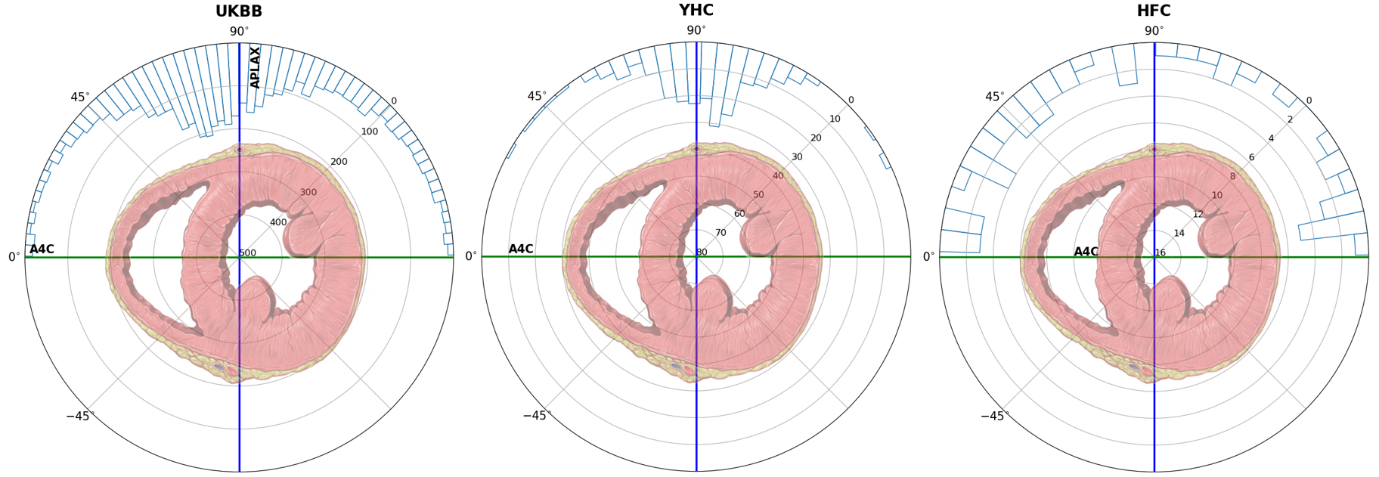


Fig. S6: Histogram visualization of the orientation angles (A_m_) for the UKBB, YHC and HFC populations respectively. The overlaid schematic represents the cross-section of the LV-RV at the papillary muscle level. $A_{m}=0^{\circ}$ coincides with the major axis aligned with the A4C view. Similarly, $A_{m}=90^{\circ}$ corresponds to the major-axis alignment with the A3C view, and $A_{m}=45^{\circ}$ with the center of mass of the aortic valve.


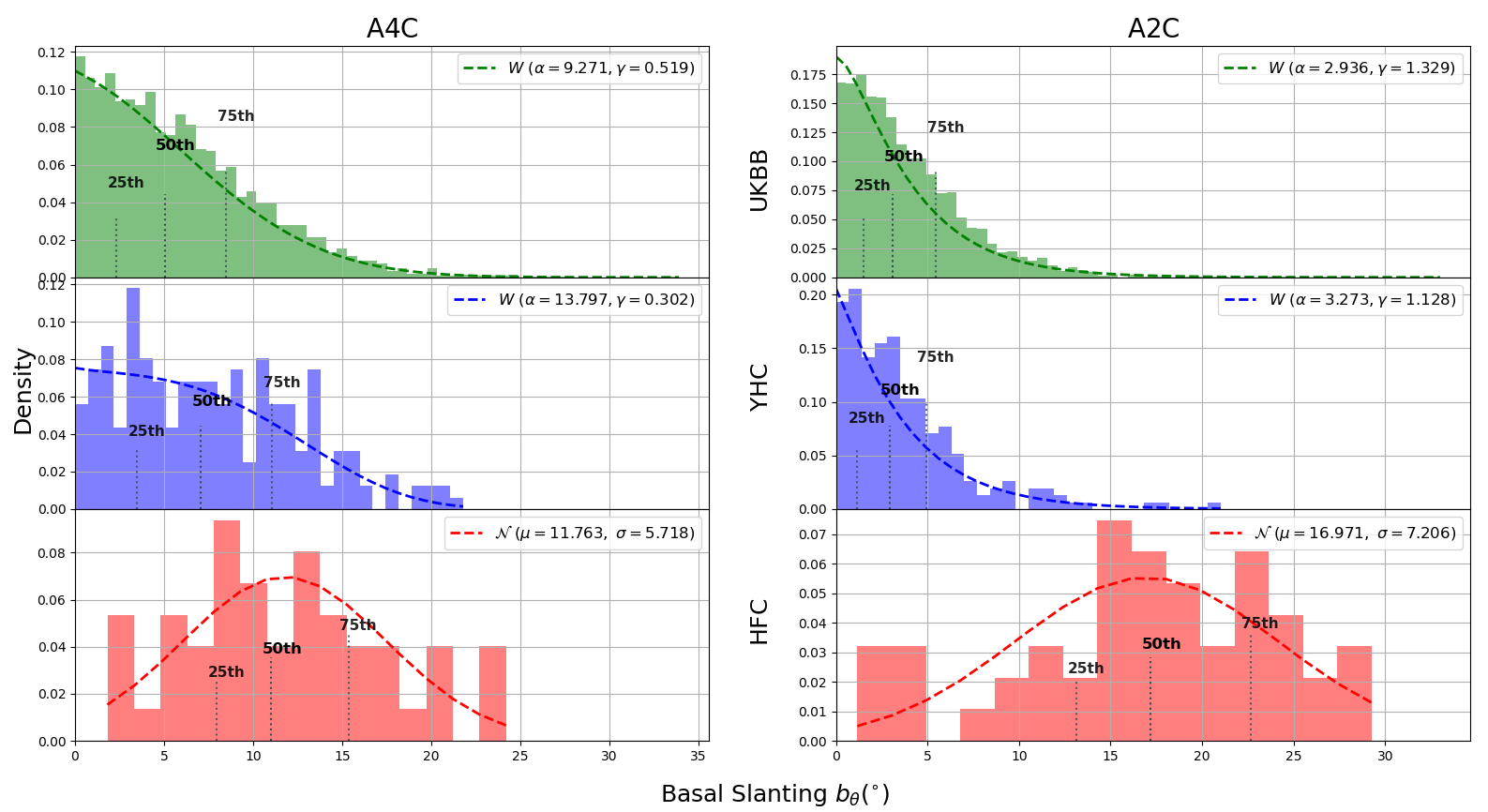


Fig. S7: Histogram of the basal slanting angles across all three populations. A Weibull distribution with decay scale parameter α and shape parameter γ is fitted to the UKBB and YHC populations and a normal distribution with mean µ and standard deviation σ is fitted to the HFC population. Note that the HFC was reconstructed from full 3D MRI acquisitions, and that the other two cohorts from a SAX MRI stack that will truncate the base.


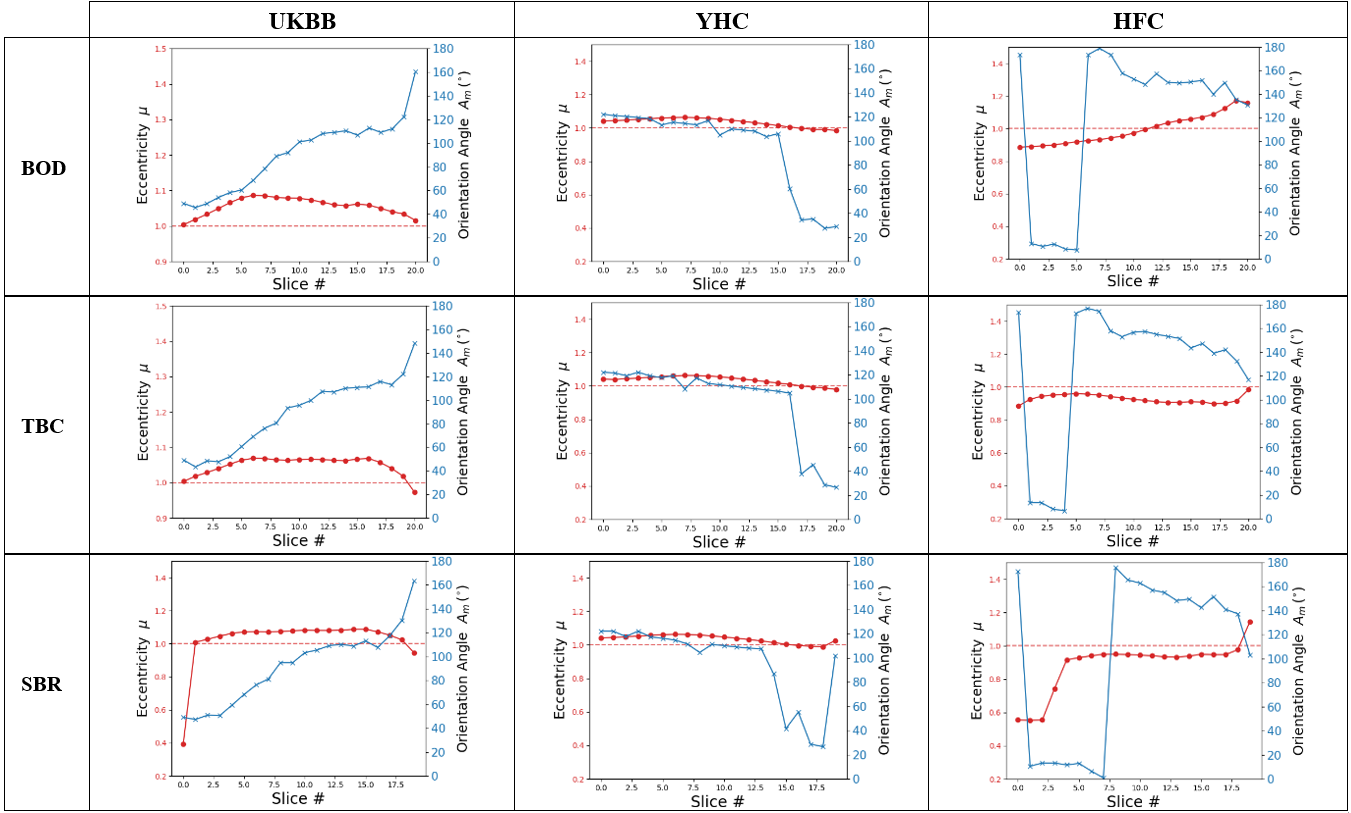


Fig. S8: Eccentricity and orientation angle profiles of exemplary cases in each population and for each method. Slice number 0 corresponds to the basal slice.


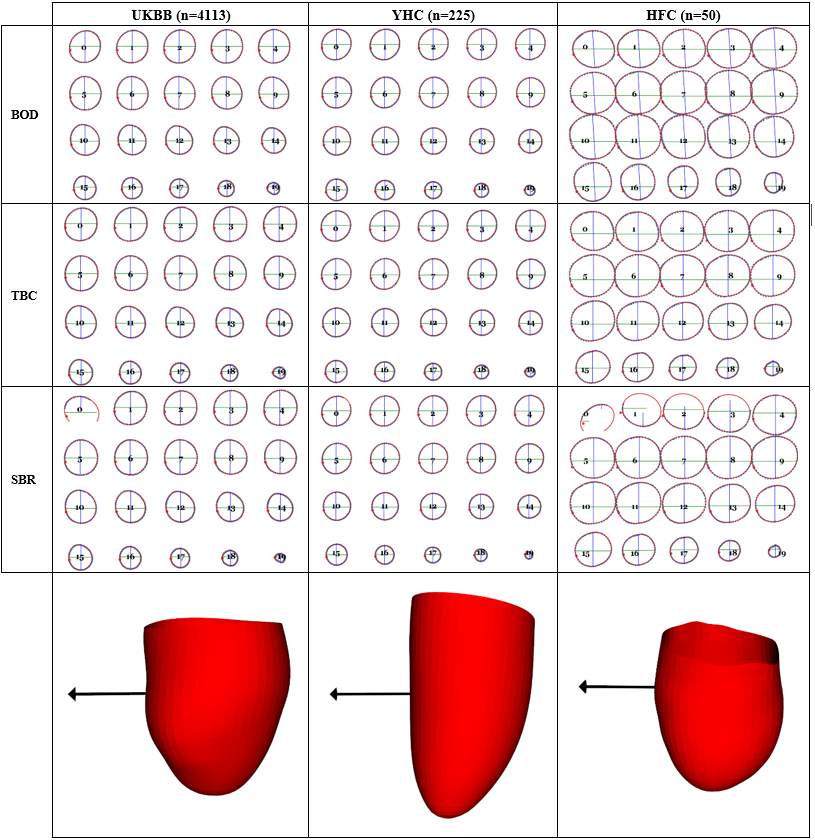


Fig. S9: LV anatomies and cross-sectional slices corresponding to eccentricity profiles in Fig. S7. Green line = A4C view, blue line = A2C view, red dot = RV direction. The black arrows in the 3D mesh visualizations represent the RV direction.

##
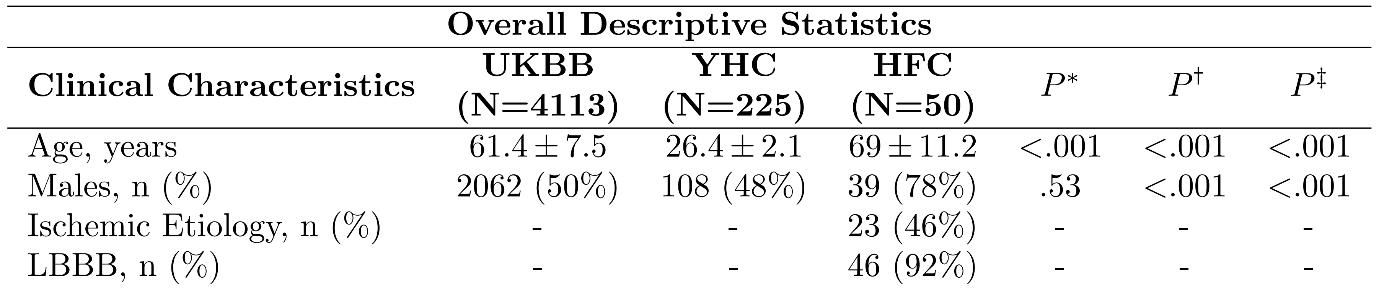
Population Baseline Characteristics

Supplementary Table 1 (TabS1): Clinical characteristics of the UKBB, YHC and HFC cohorts. LBBB; left bundle branch block. Data are shown as mean ± standard deviation, unless otherwise stated, and statistical comparison derived from Pearson chi-squared test for categorical variables or two-sample t-test for continuous variables.

** UKBB versus YHC.*

*† UKBB versus HFC.*

*‡ YHC versus HFC.*

##
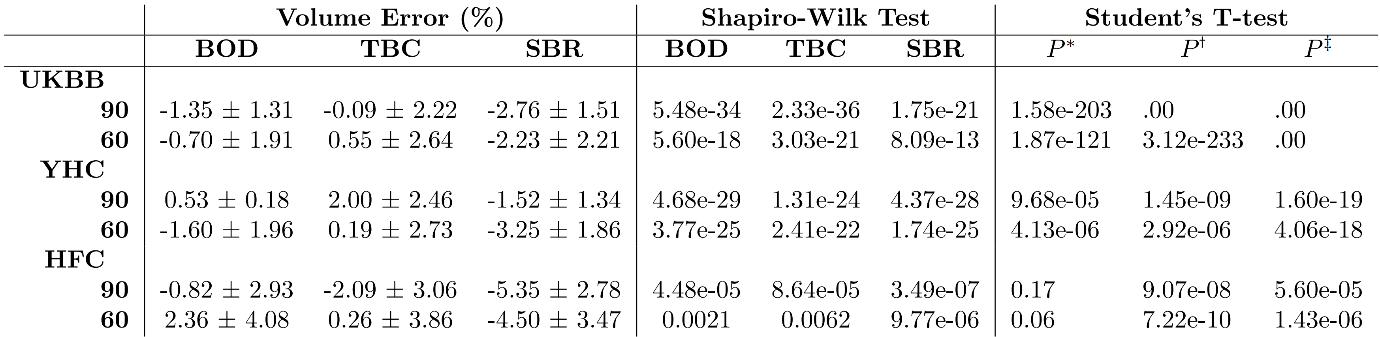
Statistical Analysis

Supplementary Table 2 (TabS2): Statistical analysis of left ventricular volume errors estimated using BOD, TBC and SBR for all cohorts. Data are shown as mean ± standard deviation, unless otherwise stated. Normality of variables was assessed using the Shapiro-Wilk test and by visual assessment of normality curves. Comparison between Simpson’s methods was performed with a 2-sided, independent-samples Student’s T-test.

** BOD versus TBC.*

*† BOD versus SBR.*

*‡ TBC versus SBR.*
